# Tangeretin alleviates sepsis-induced acute lung injury by inhibiting ferroptosis of macrophage via Nrf2 signaling pathway

**DOI:** 10.1186/s13020-025-01063-8

**Published:** 2025-01-15

**Authors:** Hui Zhang, Yan Wang, Shenghua Wang, Xiaomei Xue, Kai Huang, Dunfeng Xu, Lai Jiang, Siyuan Li, Yunqian Zhang

**Affiliations:** Department of Anesthesiology and Surgical Intensive Care Unit, Xinhua Hospital, Shanghai Jiaotong University School of Medicine, 1665 Kongjiang Road, Shanghai, 200092 China

**Keywords:** Tangeretin, Sepsis, Acute lung injury, Ferroptosis, Nrf2

## Abstract

**Background:**

Sepsis-induced acute lung injury (ALI) is a severe clinical condition accompanied with high mortality. Tangeretin, which is widely found in citrus fruits, has been reported to exert antioxidant and anti-inflammatory properties. However, whether tangeretin protects against sepsis-induced ALI and the potential mechanisms remain unclear.

**Methods:**

We established an ALI model via intraperitoneally injected with 5 mg/kg lipopolysaccharides (LPS) for 12 h. Tangeretin was applied intraperitoneally 30 min before LPS treatment. Dexamethasone (Dex) was used as a positive control. Hematoxylin and eosin (HE) staining and protein content in bronchoalveolar lavage fluid (BALF) were determined to detect the degree of lung injury. RNA-seq was also applied to explore the effect of tangeretin on ALI. In vitro, RAW264.7 were treated with Nrf2 siRNA, the expression of ferroptosis-associated biomarkers, including glutathione peroxidase 4 (GPX4) and prostaglandin-endoperoxide synthase 2 (PTGS2) were assessed. Glutathione (GSH), malondialdehyde (MDA) levels, reactive oxygen species (ROS) and inflammatory factors were also determined both in vivo and in vitro. Furthermore, mice were treated with an Nrf2 inhibitor (ML385) to verify the mechanism of tangeretin in inhibiting sepsis-induced lung injury and ferroptosis. Data were analyzed using one way analysis of variance or two-tailed unpaired t tests.

**Results:**

Our study demonstrated that tangeretin significantly alleviated lung injury, reversed the LPS-induced reduction in GPX4 and GSH, and mitigates the elevation of PTGS2 and MDA levels. Tangeretin also reduced 4-HNE and iron levels. Besides, the levels of LPS-stimulated inflammatory factors IL-6, IL-1β and TNF-α were also decreased by tangeretin. RNA-seq and bioinformatics analysis demonstrated that tangeretin inhibited inflammatory response. Mechanistically, we identified that tangeretin inhibited the GPX4-dependent lipid peroxidation through activation of Nrf2. The silence of Nrf2 abolished the inhibitory effect of tangeretin on oxidative stress, inflammatory response and ferroptosis in RAW264.7 cells. Additionally, all the protective effects of tangeretin on ALI were abolished in Nrf2 inhibitor-treated mice.

**Conclusion:**

We identified that ferroptosis as a critical mechanism contributing to sepsis-induced ALI. Tangeretin, a promising therapeutic candidate, effectively mitigates ALI through inhibiting ferroptosis via upregulating Nrf2 signaling pathway.

**Supplementary Information:**

The online version contains supplementary material available at 10.1186/s13020-025-01063-8.

## Introduction

Sepsis is often accompanied with multiple organ dysfunction due to uncontrolled host response to infection [1]. The lung is the most vulnerable organ of multiple organ dysfunction syndrome (MODS) [2]. Despite efforts have been made to improve the prognosis of patients with sepsis-induced acute respiratory distress syndrome (ARDS), it remains a critical clinical syndrome with a high mortality rate surpassing 40% [3]. Current advances in the treatment of sepsis-induced ARDS are primarily limited to lung-protective mechanical ventilation strategies and fluid resuscitation [4]. However, effective pharmacological therapies specifically targeting ARDS are yet to be identified [5]. Consequently, it is urgent to delve into the specific mechanisms of ARDS and formulate efficient treatment approaches.

Ferroptosis is defined by its reliance on iron for lipid peroxidation [6]. Distinct from apoptosis and necrosis, ferroptosis involves the build-up of lipid reactive oxygen species (ROS), an excess of intracellular iron, a reduction in glutathione (GSH) levels, the deactivation of glutathione peroxidase 4 (GPX4), and disturbances in the redox system, leading to the disintegration of the membrane and the eventual release of cell components [7, 8]. The significance of ferroptosis in sepsis-induced acute lung injury (ALI) has been highlighted in recent years [9–11]. The effectiveness of ferroptosis inhibitors, such as ferrostatin-1, has been reported to rescue lung histological changes and provide therapeutic benefits in lipopolysaccharides (LPS)-induced ALI [12]. Moreover, macrophages, which are integral to the innate immune system, play a vital role in initiating and resolving inflammatory responses, and inhibiting macrophage ferroptosis significantly alleviate sepsis-induced ALI [13, 14]. Consequently, targeting macrophage ferroptosis is a promising strategy for treating ALI.

Tangeretin, a polymethoxylated flavonoid abundant in tangerine and citrus fruits, is known for its diverse pharmacological properties and multifaceted biological effects. Recent studies have highlighted the antioxidant, neuroprotective, anti-apoptotic, and anti-inflammatory activities of tangeretin [15–18]. Specifically, tangeretin mitigated sepsis-induced myocardial dysfunction by inhibiting myocardial autophagy via the PTEN/AKT/mTOR axis [19]. While, it has been reported that tangeretin attenuated LPS-induced ALI by suppressing Th17 cell responses [20] and inhibiting ROS-dependent NLRP3 inflammasome activation [21]. Its specific regulatory effects on macrophage ferroptosis in the context of sepsis-induced ALI and the underlying mechanisms have not yet been fully elucidated.

Nuclear factor erythroid 2-related factor 2 (Nrf2) serves as a pivotal transcription factor that modulates cellular antioxidant response, has been reported to protect against various oxidative stress-related cell death including ferroptosis [22]. Nrf2 has been reported to reduce levels of ROS, therefore hindering ferroptosis and mitigating ALI caused by different etiologies [23–25]. These finding suggest that Nrf2 activation may provide a new therapeutic strategy for ALI. Furthermore, tangeretin is capable of moving Nrf2 from the cytoplasm to the nucleus in HEK293T cells [26], it also inhibits fungal ferroptosis to suppress rice blast [27]. Therefore, we hypothesize that tangeretin exerts pulmonary protection by elevating Nrf2 expression, consequently upregulating GPX4 and diminishing ferroptosis. The aim of this research is to elucidate tangeretin's efficacy as a potential novel therapeutic agent in sepsis-induced ARDS, thus providing a comprehensive understanding of its underlying mechanisms.

## Materials and methods

### Animals and experimental design

Male Institute of Cancer Research (ICR) mice (6–8 weeks) were provided by Shanghai SLAC Laboratory Animal Co., Ltd. (Shanghai, China). These mice were raised in specific pathogen-free (SPF) conditions, with environmental control set to a temperature of 22 ± 2 ℃ alongside a 12-h light/dark cycle, and were given unrestricted access to food and water. Every protocol involving animals was meticulously conducted strictly following the guidelines set by the Ethical Committee of Xinhua Hospital, affiliated to Shanghai Jiaotong University School of Medicine (approval no. XHEC-NSFC-2023-076). To induce a septic lung injury model, an intraperitoneal injection of LPS extracted from Escherichia coli O111:B4 (cat.no. L2630, Sigma-Aldrich, USA) was given at a dosage of 5 mg/kg. Tangeretin was injected intraperitoneally at 50 mg/kg, 30 min before the LPS injection. The dosages were selected in accordance with previous studies [20, 28]. Dexamethasone (Dex) (5 mg/kg, Cisen Pharmaceutical Co., Ltd. Jining, China) was used as a positive control. Furthermore, the Nrf2 inhibitor ML385 (30 mg/kg, cat.no. HY-100523, MCE Corporation, USA) was administered intraperitoneally 2 h before LPS treatment [29]. All animals were euthanized after 12 h post-LPS administration, lung tissues and bronchoalveolar lavage fluid (BALF) were harvested. For histological assessments, the left lobes of lungs were immersed in 4% paraformaldehyde, while the remaining lobes were preserved at − 80 °C for further analyses. Mice were randomly divided into groups according to the experimental requirements (n = 6 for each group).

### Structure and sources of tangeretin

Tangeretin (cat.no. HY-N0133, MCE Corporation, USA) has a molecular weight of 372.37 and a molecular formula of C_20_H_20_O_7_, with a purity of 99.27%. The chemical structure is illustrated below.
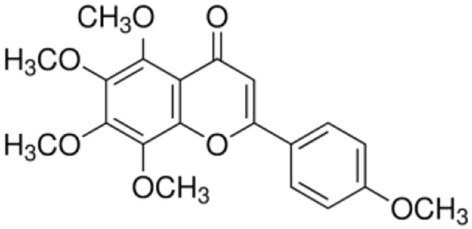


### BALF collection

BALF was collected through a tracheal catheter, employing 1 mL PBS for three successive washes. The samples were then centrifuged (4 °C, 1500 rpm, 10 min). The total protein concentration in the supernatant was subsequently measured using a BCA protein assay kit (Beyotime, Nangjing, China).

### Histopathological analysis

The left lower lobes of the lung were preserved in 4% paraformaldehyde before embedding in paraffin. Subsequently, sections of 4 μm thickness were prepared and underwent staining with hematoxylin and eosin (H&E). Two independent pathologists, specializing in lung pathology and working under blinded conditions, assessed the total surface area of each slide. The evaluation of lung inflammation was conducted according to specified criteria [30] as previously described.

### Lung wet/dry (W/D) ratio measurement

Lung samples were collected after 12 h post-LPS administration, the right upper lungs of the mice were weighed after removal, and then dried in an oven at 60 °C for 48 h for dry weight measurement. Tissue edema was calculated by measuring the W/D ratio.

### Cell culture and treatment

Murine macrophage RAW264.7 cells, purchased from Procell Life Science & Technology Co., Ltd. (Wuhan, China), were maintained in compliance with the guidelines provided by the manufacturer. The cells were pretreatment with or without tangeretin (25 μM) [20, 31] for 30 min and subjected to LPS (1 μg/mL) for 12 h. Subsequently, RAW264.7 cells and their supernatants were collected for the next experiment.

### siRNA transfection

To inhibit the gene expression, Nrf2 siRNA (cat.no. sc-37049, Santa Cruz, USA) was transfected into RAW264.7 cells using Xfect siRNA transfection reagent (Takara Bio, USA), in compliance with the manufacturer’s protocols [32]. The control siRNA consisted of a non-targeting scrambled sequence. 48 h post-transfection, Nrf2 protein expression level was analyzed, and the efficiency of transfection was assessed via western blot analysis. Cells exhibiting successful transfection were then subjected to experimental treatments as required.

### Nuclear and cytoplasmic protein extraction

The nuclear and cytoplasmic protein extraction were conducted by following the manufacturer’s instructions of the commercial kit (cat.no. P0028, Beyotime).

### Western blot analysis

Mice lung tissue and cell lysates were prepared using cold RIPA buffer (Beyotime) enriched with protease and phosphatase inhibitors. The protein levels were quantified using the BCA protein assay technique. Following, proteins (30 μg per sample) was subjected to separation by SDS-PAGE and transferred onto PVDF membranes sourced from Millipore Corp, USA. Subsequently, these membranes were treated with 5% skim milk as a blocking agent for 2 h at ambient temperature, and then they were exposed to primary antibodies targeting GPX4 (cat.no. ab125066, Abcam), prostaglandin-endoperoxide synthase 2 (PTGS2, cat.no. 12282s, Cell Signaling Technology, CST), Nrf2 (cat.no. GB113808, Servicebio Technology), heme oxygenase-1 (HO-1, cat.no. GB12104, Servicebio Technology), Kelch-like ECH-associated protein 1 (Keap 1, cat.no. GB113747, Servicebio Technology), NLRP3 (cat.no. 15101S, CST), Caspase-1 (cat.no. A0964, ABclonal), GSDMD (cat.no. ab209845, Abcam), p-MLKL (cat.no. 37333, CST), RIP1 (cat.no. ab300617, Abcam), RIP3 (cat.no. 95702, CST), Bcl-2 (cat.no. ab182858, Abcam), Bax (cat.no. ab53154, Abcam), PCNA (cat.no.13110T, CST) and β-actin (cat.no. A1978, Sigma-Aldrich) overnight at 4 °C. Following three washes with TBST, the membranes underwent incubation with horseradish peroxidase (HRP)-linked secondary antibody (Proteintech, USA) for 1 h at room temperature (RT). The detection of immunoreactive bands was carried out using an enhanced chemiluminescence (ECL) kit provided by Thermo Fisher Scientific, USA, followed by quantitative analysis with ImageJ Software version 1.8.0.

### Immunohistochemistry and immunofluorescence analysis of lung samples

Lung tissue slices (4 µm) were rehydrated and underwent antigen retrieval via heating in citrate buffer (pH 6.0). Post-retrieval, these sections were incubated with 10% bovine serum albumin (BSA) for 1 h at room temperature to block non-specific binding. After this incubation, the lung sections were incubated with primary antibodies specific for 4-HNE (cat.no. bs-6313R, Bioss Antibodies, Beijing, China). Macrophage activation was detected by immunofluorescence through primary antibodies against F4/80 (cat.no. GB11027, Servicebio Technology) throughout the night at 4 °C. This was succeeded by a 1 h incubation at 37 °C with a cyanine 3 (CY3)-conjugated goat anti-rabbit secondary antibody (cat.no. GB21303, Servicebio Technology) conducted in darkness. The nuclei were then counterstained using DAPI (cat.no. C1005, Beyotime). Images were captured using an Olympus fluorescence microscope.

RAW264.7 macrophage cells from each experimental group were stabilized using 4% paraformaldehyde for a duration of 15 min, made permeable with 0.3% Triton X-100, and then blocked using 5% BSA for half an hour. After blocking, the cells underwent incubation overnight at 4 °C with an Nrf2 antibody (cat.no. GB113808, Servicebio Technology), followed by incubated with CY3-labeled goat anti-rabbit secondary antibody at RT. Then nuclei were stained with DAPI, and the fluorescent images were obtained using an Olympus fluorescence microscope.

### Quantification of cytokines and detection of tissue iron

The concentrations of cytokines IL-6, IL-1β, and TNF-α in lung tissues and cell supernatants were accurately quantified utilizing specific ELISA kits (cat.no. E-EL-M0044, E-EL-M0037, E-EL-M3063, respectively; Elabscience, China), adhering closely to the protocols outlined by the manufacturer. The tissue iron concentrations were also determined as instructed (cat.no. A039-2-1, Nanjing Jiancheng Bioengineering Institute, Nanjing, China).

### Measurement of myeloperoxidase (MPO) activity, malondialdehyde (MDA) and reduced GSH content

Neutrophil infiltration was quantitatively evaluated through the measurement of MPO activity in lung tissues using a designated assay kit (cat.no. A044-1-1, Nanjing Jiancheng Bioengineering Institute), in strict compliance with the manufacturer's guidelines. Additionally, the biomarkers of membrane lipid peroxidation and non-enzymatic antioxidant capacity, MDA and reduced GSH, were quantified in both lung tissues and cell supernatants using relevant colorimetric assay kits (cat.no. E-BC-K025-M, E-BC-K030-M, Elabscience, China), as previously described [33].

### Detection of intracellular ROS production

To monitor intracellular ROS accumulation, fluorescence probe dihydroethidium (DHE) was employed. Following the treatment, RAW264.7 cells were incubated for 30 min at 37 °C in a light-deprived environment with DHE (10 µM, cat.no. S0063, Beyotime) following the manufacturer's protocol. The cells were then rinsed twice with PBS before images were acquired utilizing an Olympus fluorescence microscope.

### RNA-sequencing (RNA-seq) analysis

Lung tissue specimens from the groups treated with LPS alone and LPS combined with tangeretin (Tan) were analyzed using RNA sequencing by OE Biotech Co., Ltd. in Shanghai, China. The procedure began with the extraction of total RNA using TRIzol reagent (Invitrogen, USA), followed by assessment of RNA purity and concentration. RNA integrity was assessed with the Agilent 2100 Bioanalyzer (Agilent Technologies, USA). Subsequently, cDNA libraries were constructed utilizing the VAHTS Universal V6 RNA-seq Library Prep Kit, followed by sequencing performed on an Illumina Novaseq 6000 platform. Each sample yielded around 50 million clean reads. Differentially expressed genes (DEGs) were determined based on a *p*-value < 0.05 and |Log_2_FC|> 1. Functional annotation and pathway analyses were performed using gene set enrichment analysis (GSEA) through the GSEA software. The sequence data obtained have been submitted to the NCBI Gene Expression Omnibus (GEO) database and are available under the accession number GSE252755.

### Statistical analysis

The Data are presented as means ± standard error of the means (SEM). For the comparison of two distinct groups, a two-tailed unpaired Student's t-test was applied. In the case of multiple group comparisons, analysis of variance (ANOVA) was utilized, followed by a Student–Newman–Keuls post-hoc analysis, utilizing SPSS Statistics 26.0 software. Statistical significance was established at a *p*-value of less than 0.05.

## Results

### Tangeretin mitigates sepsis-induced ALI and inflammatory infiltration in lung

**Fig. 1:**
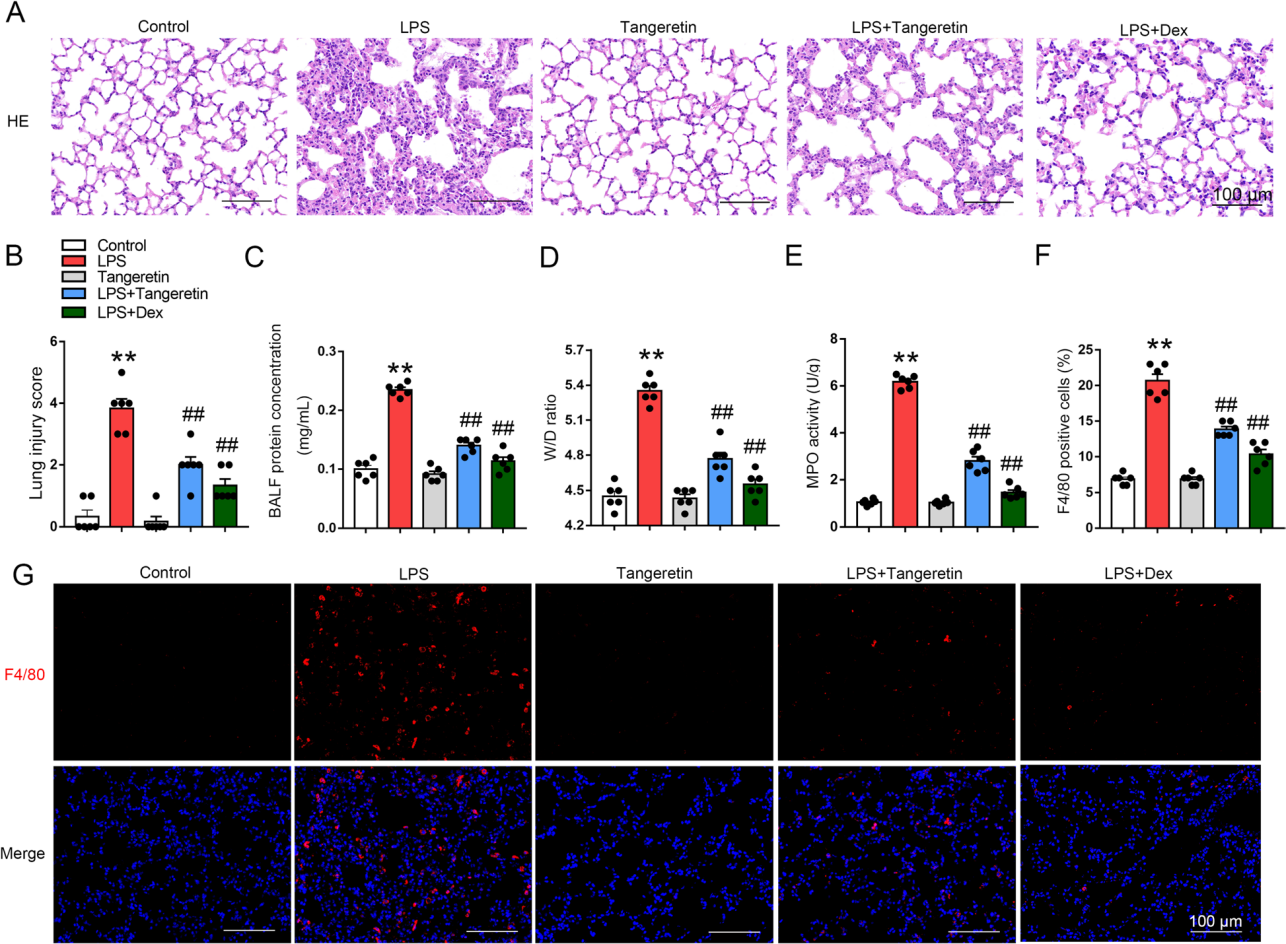
Tangeretin alleviates sepsis-induced ALI and inflammatory infiltration in lung. Mice were administered with tangeretin and subsequent exposed to LPS challenge for 12 h. **A** Lung tissue morphology was examined using HE staining. **B** The extent of lung injury was quantified. **C** Protein concentration of BALF and (**D**) Lung W/D ratio was determined as indexes of pulmonary edema. **E** MPO activity in lungs were assessed. **F**, **G** Lung sections showed F4/80 positive cells identified by immunofluorescence staining (nuclei in blue, F4/80 in red), and the percentages of F4/80^+^ cells were quantified. Original magnification, × 200. Scale bar, 100 μm. Data are presented as the mean ± SEM (n = 6). **p < 0.01 vs. Control group, ##*p* < 0.01 vs. LPS group

To address the impact of tangeretin on sepsis-induced ALI, LPS and tangeretin were injected intraperitoneally. Dex was used as a positive control. Not surprisingly, tangeretin and Dex significantly suppressed LPS-induced pathological changes including decreased interstitial edema and inflammatory cell infiltration (Fig. 1A, B). Tangeretin and Dex-treated mice had a lower protein content in the BALF than mice challenged with LPS without tangeretin (Fig. 1C). Meanwhile, LPS-induced a higher W/D weight ratio compared with control group, which was markedly improved in tangeretin and Dex-treated mice (Fig. 1D). Subsequently, we measured the activity of MPO, an indicator of neutrophil presence, and noted a markedly rise in MPO levels in the group exposed to LPS compared to the untreated control group. Whereas, the administration of tangeretin and Dex mitigated the elevation in MPO levels induced by LPS treatment (Fig. 1E). In addition, F4/80, a biomarker for macrophage infiltration, was used to explore the role of tangeretin in ALI. In the lung tissues of the control group, there were very few F4/80-positive cells. However, mice subjected to LPS developed severe infiltration of F4/80^+^ macrophage and this was alleviated by treatment with tangeretin and Dex (Fig. 1F, G). To investigate whether tangeretin influences macrophage polarization, we conducted dual fluorescence staining to identify M1 macrophages (F4/80⁺iNOS⁺) and M2 macrophages (F4/80⁺CD206⁺). In addition, we assessed the mRNA expression levels of pro-inflammatory markers (TNF-α, IL-1β, and IL-6) and anti-inflammatory markers (Arg1, IL-10, and CD206) in lung tissue. Results showed that tangeretin appears to favor the polarization of macrophages toward the M2 phenotype, characterized by increased proportion of F4/80⁺CD206⁺ cells (Fig. S1A) and elevated expression of anti-inflammatory markers (Arg1, IL-10, and CD206) (Fig. S1B&D). Conversely, we observed a decreased proportion of F4/80⁺iNOS⁺ cells (Fig. S2A) and reduced levels of pro-inflammatory markers (iNOS, TNF-α, and IL-6) in lung tissue (Fig. S2B&D). Collectively, the findings indicate that tangeretin substantially mitigates the severity of sepsis-induced ALI and reduces inflammation infiltration in mice.

### Tangeretin suppresses ferroptosis in murine lung tissue during sepsis-induced ALI

**Fig. 2:**
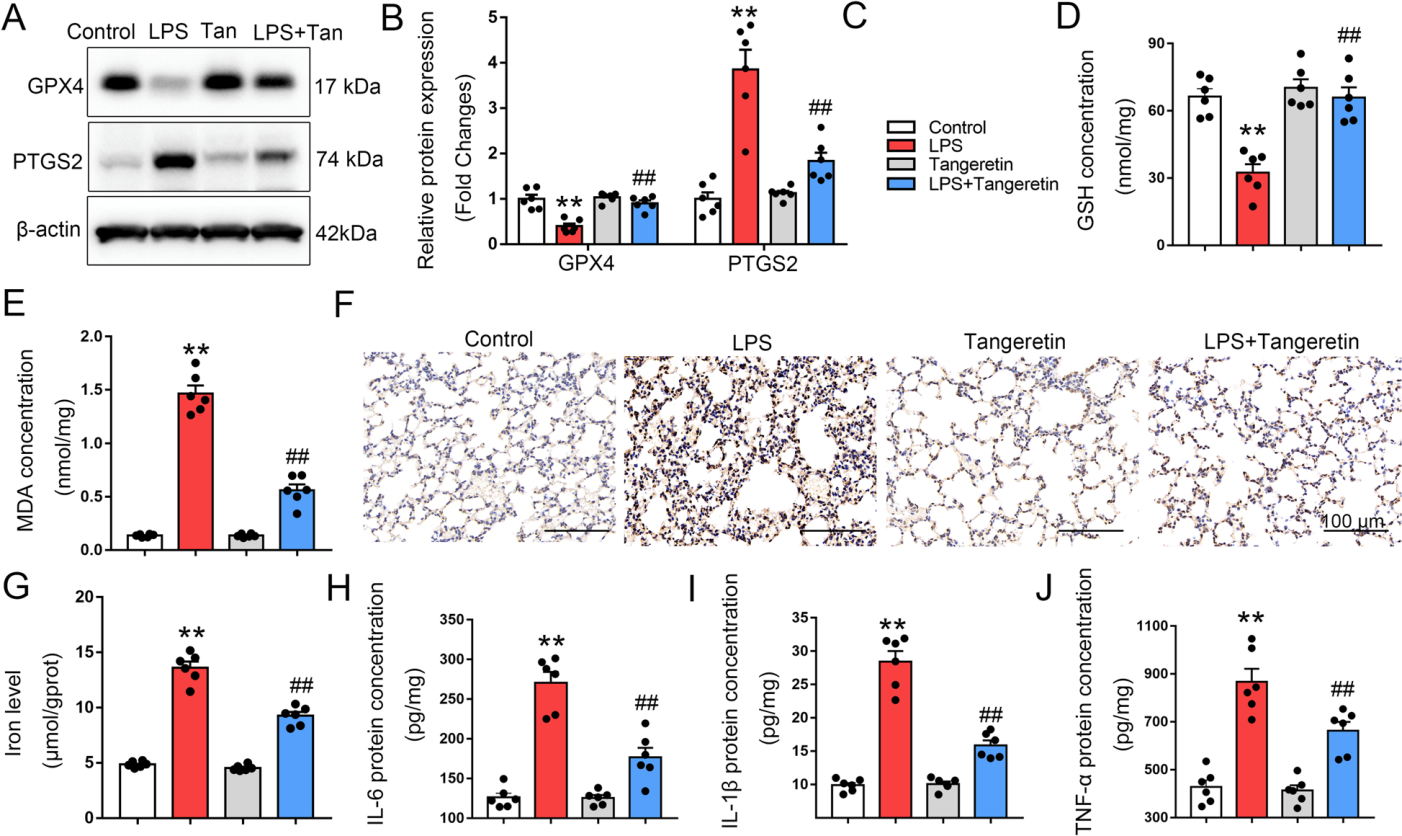
Tangeretin inhibits ferroptosis in murine lung during sepsis-induced ALI. Mice received tangeretin treatment followed by a 12 h exposure to an LPS challenge. The expression levels of GPX4 and PTGS2 in lung tissues were evaluated semi-quantitatively by western blotting. **A**, **B** Representative bands and corresponding histogram are shown. **C** Experimental groups are distinguished by distinct color codes. Levels of (**D**) GSH, (**E**) MDA and (**G**) iron level were quantified via colorimetric assays. **F** 4-HNE was assessed by immunohistochemistry staining. **H**–**J** Cytokines IL-6, IL-1β, and TNF-α in lung tissues were determined by ELISA. Original magnification, × 200. Scale bar, 100 μm. All data are expressed as the mean ± SEM (n = 6). **p < 0.01 vs. Control group, ##*p* < 0.01 vs. LPS group

Previous research identified tangeretin, a fungal ferroptosis inhibitor derived from plants, as a promising new antifungal agent for the sustainable control of severe blast disease in vital cereal crops [27]. We then explored the relationship between tangeretin and ferroptosis. GPX4 and PTGS2 are two well-known markers of ferroptosis. We found that the expression of GPX4 was decreased and PTGS2 was elevated after LPS treatment, whereas tangeretin treatment significantly reversed the expression of GPX4 and PTGS2 (Fig. 2A, B). GSH (a reducing agent for antioxidation) and lipid peroxidation indicator MDA were also assessed. LPS exposure led to a significant decrease in GSH and an increase in MDA, while tangeretin intervention increased GSH levels and lowered MDA content in lung tissue (Fig. 2D, E). 4-hydroxy-2-nonenal (4-HNE), aldehydic metabolites from lipid peroxidation process, was also assessed. The 4-HNE (Fig. 2F) staining and iron level (Fig. 2G) confirmed that LPS can promote lipid peroxidation in lung, but tangeretin significantly inhibits it. Additionally, the administration of tangeretin significantly mitigated the inflammatory response within lung tissues, evidenced by the decreased concentrations of the cytokines IL-6, IL-1β, and TNF-α (Fig. 2H–J). In conclusion, tangeretin effectively suppressed ferroptosis in ALI triggered by sepsis in mice.

### Tangeretin activates Nrf2 signaling pathway in vivo* during sepsis-induced ALI*

**Fig. 3:**
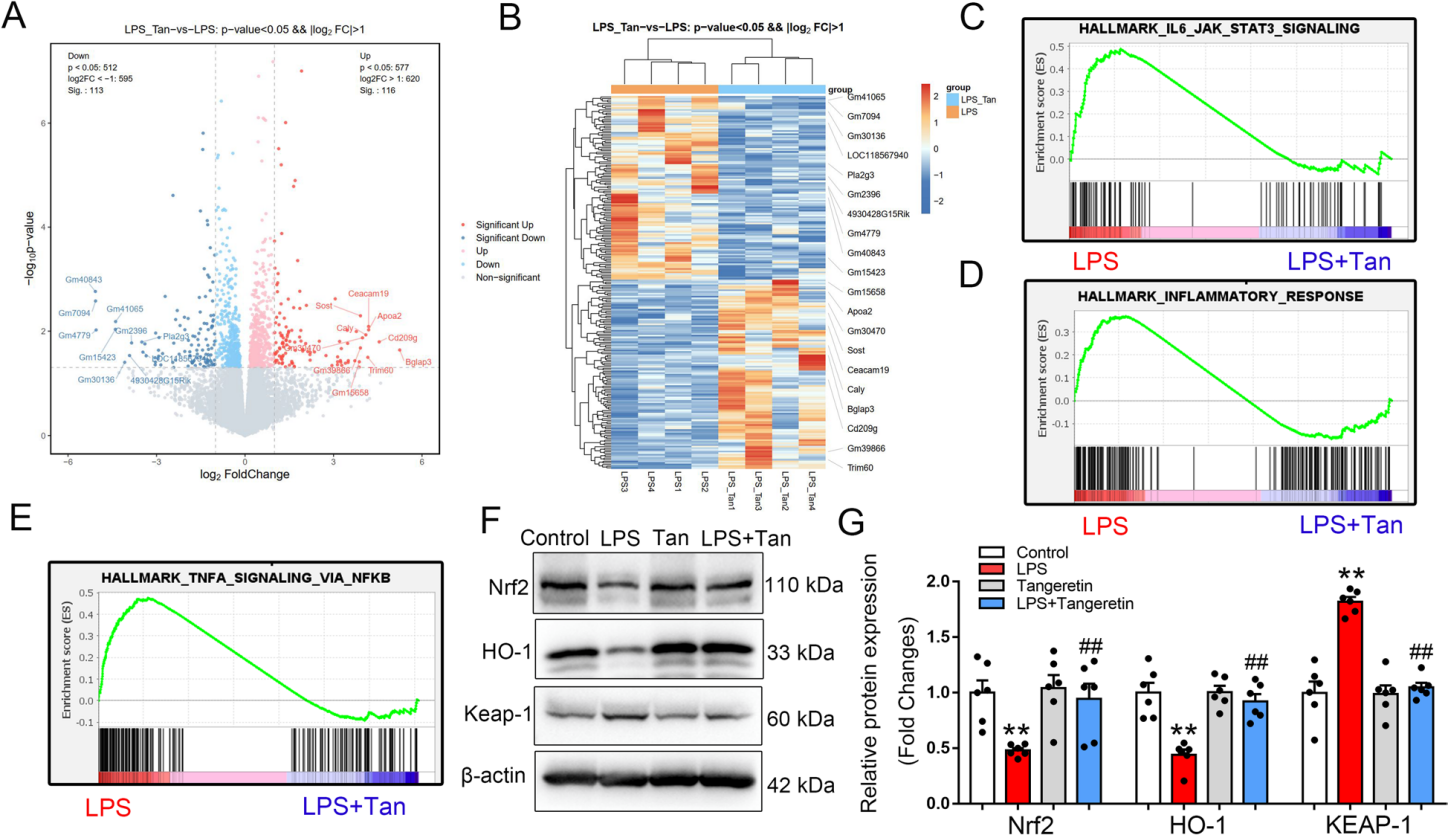
Tangeretin activates Nrf2 signaling pathway in vivo during ALI. Mice underwent treatment with tangeretin and then subjected to LPS for 12 h. Differential gene expression in lung tissues from the LPS and LPS + Tan groups was elucidated through RNA-seq. **A**, **B** Volcano plot and heatmap illustrated the clustering of differentially expressed genes in LPS group vs. LPS + Tan groups (n = 4). GSEA analysis demonstrated (**C**) IL6_JAK_STAT3 SIGNALING, **D** INFLAMMATORY_RESPONSE, and (**E**) TNF-α_SIGNALING_VIA_NF-κB pathway were inhibited in the LPS + Tan group. **F** Protein expression levels of Nrf2, HO-1 and Keap-1 were examined, (**G**) histograms are shown on the right of representative bands. Statistical data are expressed as the mean ± SEM (n = 6). **p < 0.01 vs. Control group, ##*p* < 0.01 vs. LPS group

To further validate the function of tangeretin in sepsis-induced ALI, differential gene expression analysis was conducted between LPS and LPS + Tan group using RNA-seq data (*p*-value < 0.05, |Log_2_FC|> 1, Fig. 3A, B). GSEA comparing the LPS and LPS + Tan groups showed significant enrichment of pathways such as IL6_JAK_STAT3_SIGNALING, INFLAMMATORY_RESPONSE, and TNF-α_SIGNALING_VIA_NF-κB (Fig. 3C–E). These findings highlight the anti-inflammatory capabilities of tangeretin in combating sepsis-induced ALI. Nrf2 is a transcription factor that regulates the expression of antioxidant response elements (AREs) and is itself regulated by Keap1, could promote HO-1 and GPX4 to inhibit ferroptosis. Prior investigations have demonstrated that activating of Nrf2 signaling pathway inhibited ferroptosis, thus exerting protective effect on ALI [24, 29, 32, 34]. Our research revealed that tangeretin counteracted the LPS-induced increase of Keap1 and reduction of Nrf2. Moreover, tangeretin enhanced the expression of the downstream target gene HO-1 (Fig. 3F, G). Thus, tangeretin may inhibit ferroptosis by activating Nrf2 in mouse lung tissues induced by LPS.

### Tangeretin inhibits LPS-induced ferroptosis via* Nrf2-dependent pathways in macrophage*

**Fig. 4:**
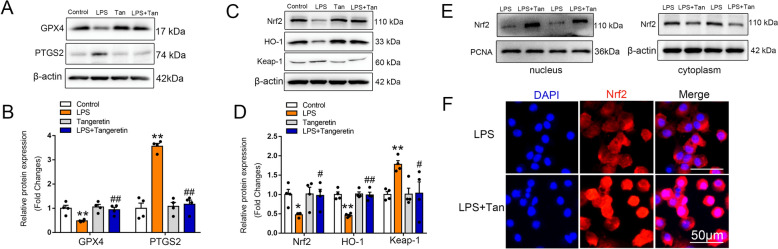
Tangeretin inhibits LPS-induced ferroptosis through Nrf2-dependent pathways in macrophage. RAW 264.7 cells pretreated with tangeretin (25 μM) were exposed to LPS for 12 h. **A**, **B** Indicative protein bands of GPX4 and PTGS2, accompanied by semi-quantitative analysis. **C** Protein expression of Nrf2 signaling pathway were examined, **D** histograms are shown below representative bands. The data are presented as the mean ± SEM (n = 4). *p < 0.05, **p < 0.01 vs. Control group, #*p* < 0.05, ##*p* < 0.01 vs. LPS group. **E** Nrf2 protein expression in nucleus and in cytoplasm were detected. **F** Immunofluorescence staining for Nrf2 (red) and nuclei (DAPI, blue) was conducted. Original magnification, × 200. Scale bar, 50 μm

**Fig. 5:**
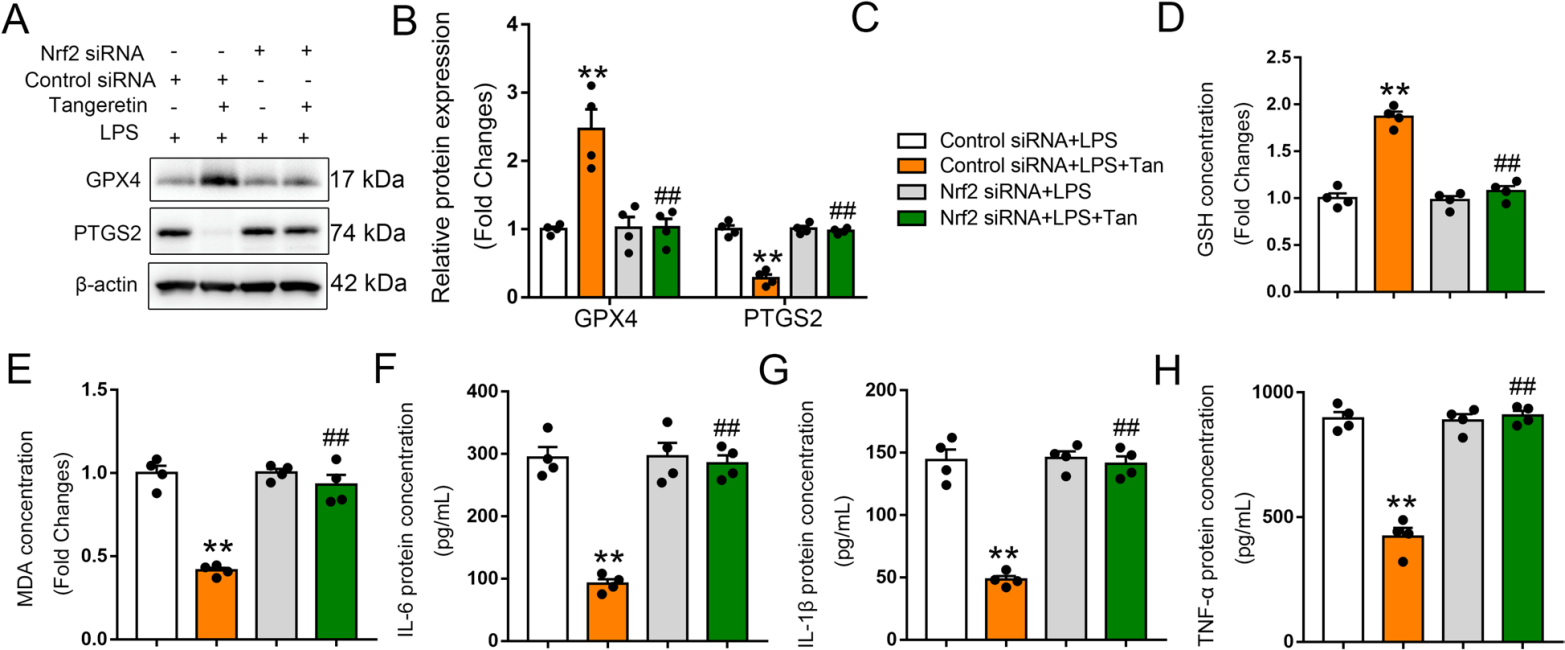
Tangeretin inhibited ferroptosis through Nrf2 activation in vitro. RAW264.7 cells transfected with control or Nrf2 siRNA for 48 h, followed by LPS/LPS + Tan treatment. **A**, **B** Western blot analysis of GPX4 and PTGS2 in RAW264.7 cells, representative bands and corresponding histograms are shown. **C** Group differentiation by color coding. **D**, **E** GSH contents and MDA levels in cell supernatants were measured. **F**–**H** IL-6, IL-1β, and TNF-α levels in cell supernatants were quantified. Statistical data are expressed as the mean ± SEM (n = 4). **p < 0.01 vs. Control siRNA + LPS group, ##*p* < 0.01 vs. Control siRNA + LPS + Tan group

Macrophage ferroptosis has been demonstrated to be involved in ALI [34]. We found that tangeretin inhibited LPS-induced ferroptosis in RAW264.7 cell (Fig. 4A, B). To verify that the protective effect of tangeretin on macrophages is primarily mediated through ferroptosis, we performed Western blot analysis to examine the effects of tangeretin on pyroptosis-, necroptosis-, and apoptosis-related proteins. The results showed that tangeretin effectively reduced the levels of pyroptosis-related proteins and significantly alleviated the expression of necroptosis- and apoptosis-related proteins (Fig. S3A–C). Furthermore, we conducted CCK8 assays to assess cell viability after the addition of different cell death pathway inhibitors. The results demonstrated that tangeretin substantially improved the viability of macrophages suppressed by LPS. However, when a ferroptosis inhibitor was added, no further improvement in macrophage viability was observed. In contrast, when pyroptosis, necroptosis, or apoptosis inhibitors were added alongside tangeretin, macrophage viability was further enhanced compared to tangeretin alone (Fig. S3D). These findings suggest that pyroptosis, necroptosis, and apoptosis may be involved in the protective effects of tangeretin but are not the most critical pathways. Ferroptosis is likely the predominant form of cell death targeted by tangeretin. Moreover, treatment of LPS-stimulated macrophage with tangeretin upregulated Nrf2 and HO-1 expression, along with a decrease in Keap 1 level (Fig. 4C, D), Nrf2 was assessed in nuclear lysates and cytoplasm, representative bands and immunofluorescence staining showed that tangeretin significantly promoted Nrf2 entry into the nucleus when RAW264.7 cell was stimulated with LPS (Fig. 4E, F). Subsequently, to further elucidate whether tangeretin alleviates ferroptosis through activating Nrf2 signaling pathway, we applied Nrf2 siRNA to knockdown the expression of Nrf2 in RAW264.7. Knockdown efficiency was verified by western blotting (Fig. S4A&B). The results confirm that Nrf2 siRNA effectively suppresses Nrf2 expression. Ferroptosis is a recently identified form of programmed cell death, distinguished by the buildup of lipid ROS, we demonstrated that Nrf2 knockdown abolished the inhibition effect of tangeretin on ROS accumulation (Fig. S4C). We have performed additional experiments using BODIPY 581/591 C11 probes to measure lipid peroxidation levels during ferroptosis. The results confirm that tangeretin significantly reduces lipid peroxidation levels, while Nrf2 siRNA reversed these effects (Fig. S4D). Furthermore, when Nrf2 was silenced, there was a notable reduction in GPX4 level and a rise in PTGS2 levels, nearly completely negating the protective effects of tangeretin (Fig. 5A, B). In contrast, Nrf2-silenced group exhibited a decrease in GSH level and an elevation in MDA level when compared with control siRNA group that exposed to LPS and tangeretin (Fig. 5D, E). Inflammatory factor such as IL-6, IL-1β and TNF-α were also reversed due to Nrf2 silencing (Fig. 5F–H). Thus, we conclude that tangeretin prevents LPS-induced ferroptosis via Nrf2-dependent pathways in macrophage.

### Tangeretin prevents ferroptosis induced by sepsis through the activation of Nrf2 in vivo

**Fig. 6:**
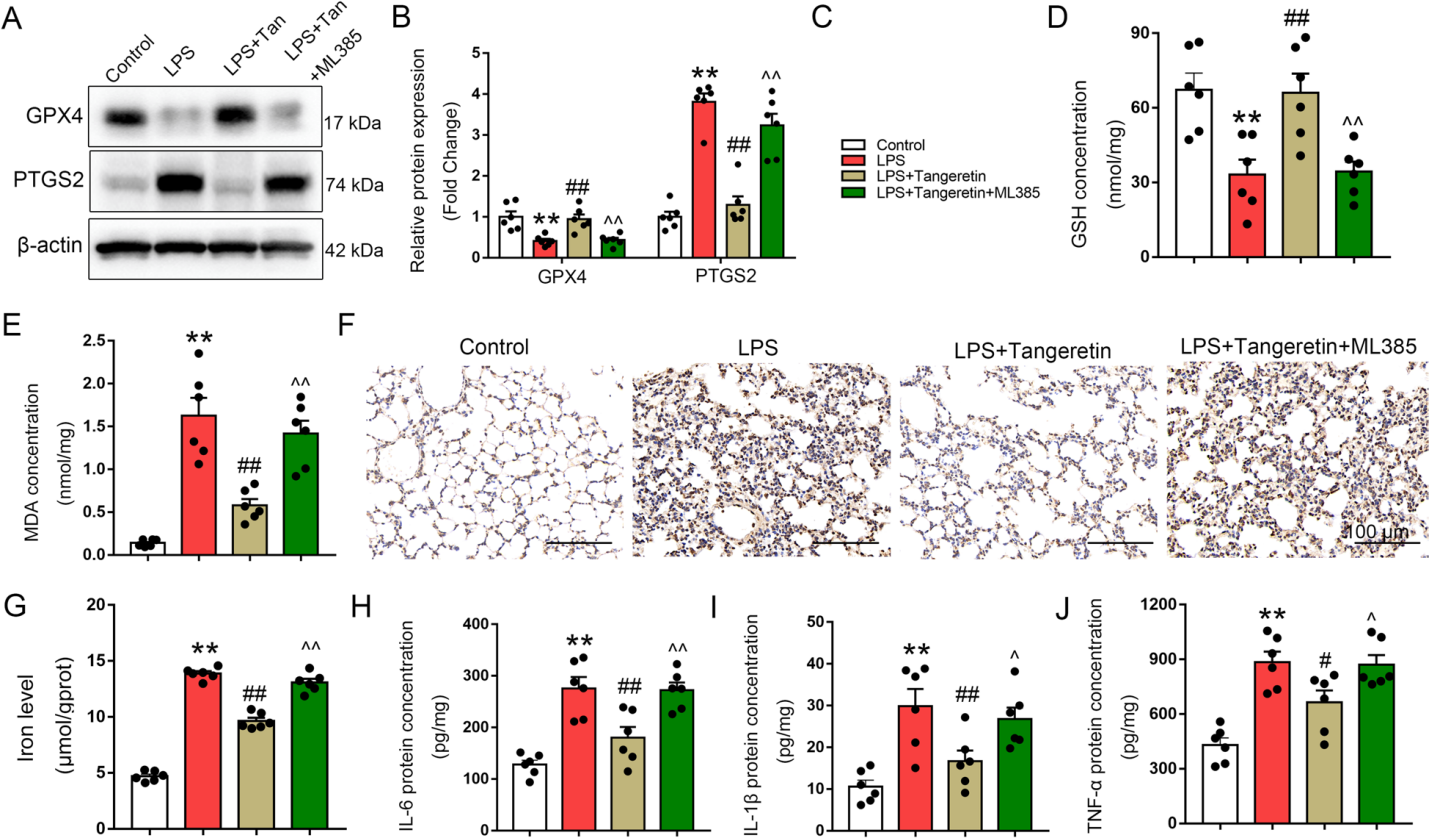
Tangeretin inhibited ferroptosis through Nrf2 activation in vivo. ML385 (30 mg/kg) was administered prior to LPS/LPS + Tan treatment. **A**, **B** Representative bands of ferroptosis-related proteins in lung tissues and corresponding histograms are shown. **C** Group differentiation by color coding. **D**, **E**, **G** The levels of GSH, MDA, iron level, along with cytokines (**H**–**I**) IL-6, IL-1β, and TNF-α in lung tissues were quantified. **F** 4-HNE was assessed by immunohistochemistry staining. Original magnification, × 200. Scale bar, 100 μm. Results are expressed as the mean ± SEM (n = 6). **p < 0.01 vs. Control group; #*p* < 0.05, ##*p* < 0.01 vs. LPS group; ^*p* < 0.05, ^^*p* < 0.01 vs. LPS + Tan group

To delve deeper into whether tangeretin exerts its anti-ferroptosis effects by activating Nrf2, mice were pre-administered with an Nrf2 inhibitor (ML385, at a dosage of 30 mg/kg) for 2 h before being challenged with LPS. As depicted in Fig. 6A, B, in the lung tissues of mice stimulated with LPS, the protein level of GPX4 was elevated and PTGS2 was diminished following treatment with tangeretin. However, ML385 administration markedly abrogated the effect of tangeretin. Moreover, tangeretin treatment increased the extent of GSH and decreased the MDA content, 4-HNE, iron level in lung tissue stimulated by LPS (Fig. 6D–G), inflammatory factors IL-6, IL-1β and TNF-α triggered by LPS were also attenuated by tangeretin supplementation, all these were reversed by pretreatment with ML385 (Fig. 6H–J). To evaluate Nrf2 nuclear translocation and its transcriptional activity in macrophages from mouse lung tissue, we performed dual immunofluorescence staining for Nrf2 and F4/80 (a macrophage marker). The results revealed that, compared to the LPS group, Nrf2 nuclear translocation in macrophages was significantly increased in the LPS + Tan group. Additionally, treatment with ML385 markedly reduced Nrf2 nuclear expression, indicating effective inhibition of Nrf2 activity (Fig. S5). These findings further support the regulatory role of tangeretin in activating Nrf2 and validate the inhibitory effect of ML385 in this context. Therefore, tangeretin inhibited ferroptosis through Nrf2 activation in lung tissues during ALI.

### Tangeretin inhibits sepsis-induce d ALI through Nrf2 activation in vivo

**Fig. 7:**
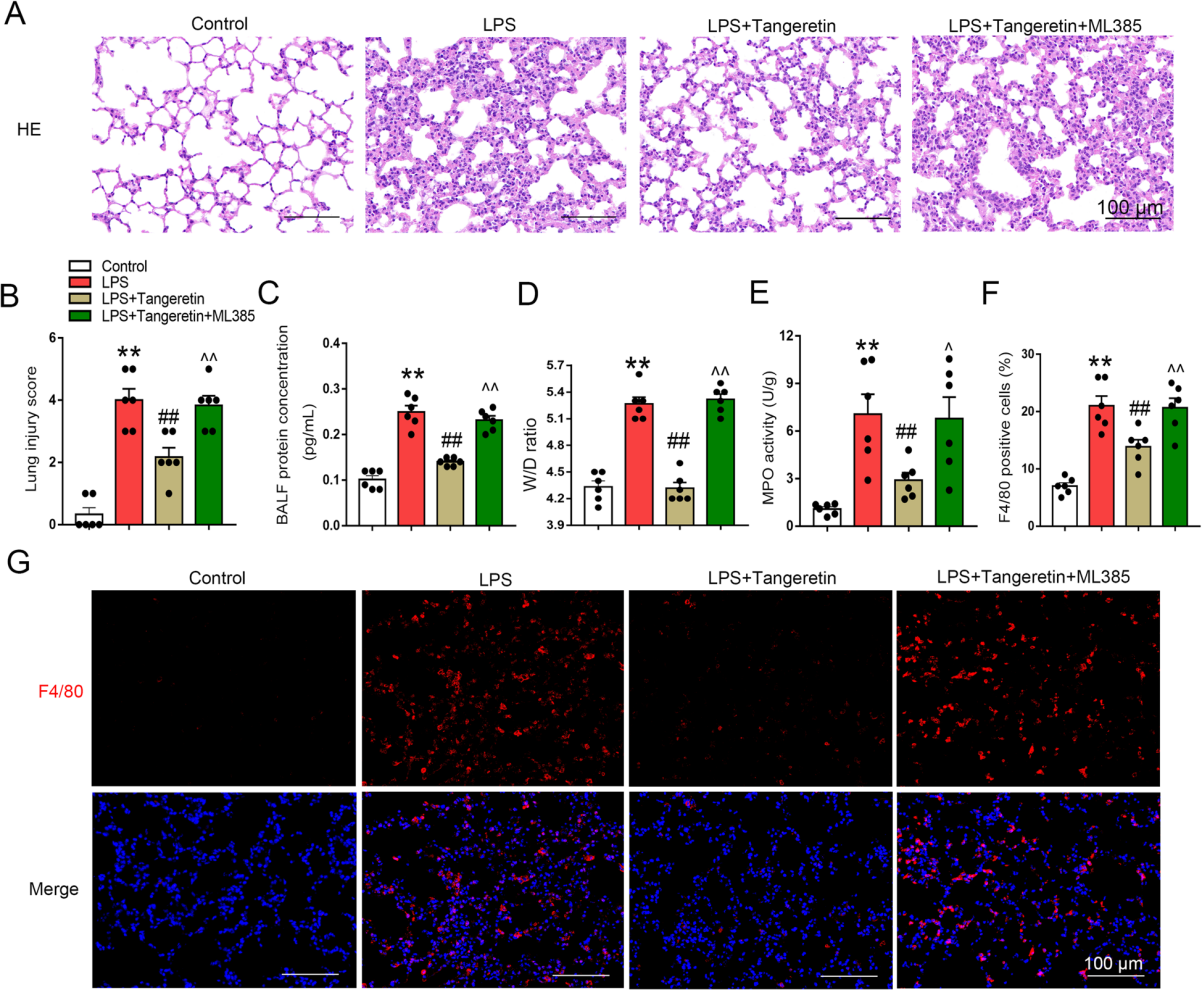
Tangeretin inhibited sepsis-induced ALI through Nrf2 activation in vivo. ML385 (30 mg/kg) was administered prior to tangeretin treatment. **A**, **B** Lung tissue sections were evaluated using HE staining and subsequent lung injury scoring. **C**, **D**, **E** Protein concentration of BALF, lung W/D ratio and MPO activity were measured. **F**, **G** F4/80 was analyzed using immunofluorescence, and the percentages of F4/80^+^ cells were quantified. Original magnification, × 200. Scale bar, 100 μm. All data are expressed as the mean ± SEM (n = 6). **p < 0.01 vs. Control group; ##*p* < 0.01 vs. LPS group; ^*p* < 0.05, ^^*p* < 0.01 vs. LPS + Tan group

Finally, to determine if the protective role of tangeretin against sepsis-induced ALI was mediated by Nrf2, corresponding experiments were conducted. As depicted in Fig. 7A, B, lung tissue damage visualized by HE staining explicitly demonstrated that pulmonary hemorrhage and thickening alveolar wall were mitigated in tangeretin-treated mice post-LPS intervention, whereas ML385 abolished the effect of tangeretin. Similarly, tangeretin decreased the protein content in the BALF and W/D ratio of LPS-exposed mice, ML385 significantly recovered the protein level in the BALF and W/D ratio (Fig. 7C, D). MPO activity, F4/80 positive cells in lung tissues were reduced by tangeretin supplementation in LPS-exposed mice, however, these changes were largely reversed when administrated with ML385 (Fig. 7E–G).

## Discussion

Sepsis-induced ALI remains a critical life-threatening condition, representing a major public health challenge due to the paucity of personalized treatment approaches [35, 36]. Consequently, new therapeutic approaches are essential for enhancing the clinical outcomes of patients suffering from ALI. In this context, our study demonstrated that tangeretin, a bioactive flavonoid extracted from orange peel [37] effectively inhibits ferroptosis, offering protection against ALI, predominantly through the activation of the Nrf2 signaling pathway. These results highlight tangeretin's promise as an innovative therapeutic option for managing sepsis-induced ALI.

Tangeretin has attracted significant attention due to its diverse biological activities. Notably, it was demonstrated to exert anti-inflammatory properties and neuroprotective impacts on microglial cells, as well as in models of cerebral ischemia–reperfusion injury in rats [38, 39]. Additionally, it mitigates cisplatin-induced kidney injury in rats via anti-inflammatory cascades and oxidative disturbances [40]. Li and colleagues noted a marked reduction in TNF-α levels in BALF and a significant decrease in MPO activity in lung tissues, highlighting tangeretin's ability to attenuate LPS-induced lung injury [20]. Furthermore, tangeretin administration significantly alleviates pulmonary edema and reduces pulmonary congestion, alveolar wall thickening, and leukocyte infiltration, while suppressing the expression of inflammatory cytokines and the infiltration of inflammatory cells in models of sepsis-induced ALI [20, 41]. Consistent with these findings, the present study demonstrated that tangeretin alleviates LPS-induced lung tissue damage, decreases MPO activity and reduces macrophage infiltration. Importantly, tangeretin also improved endothelial hyperpermeability in the lungs of LPS-induced ALI mice. Moreover, our study found that tangeretin promotes the polarization of macrophages from M1 to M2, thereby enhancing the production of anti-inflammatory mediators in LPS-induced ALI. Tangeretin treatment led to a substantial decrease in the concentrations of pro-inflammatory cytokines such as IL-6, IL-1β and TNF-α, aligning with previous research [38, 39].

Ferroptosis, a regulated form of cell death distinguished by disruptions in iron and lipid metabolism, has emerged as a significant focus in the progression of ALI and other diseases [12, 34, 42]. Inhibition of ferroptosis has been demonstrated to markedly mitigate sepsis-induced ALI, highlighting the complexity of the mechanisms initiating ferroptosis, including the dysregulation of the cystine/glutamate antiporter system (System Xc−), composed of the SLC7A11 and SLC3A2 subunits, is essential for intracellular free radical scavenging and GSH production, with its inhibition leading to decreased cellular GSH levels and increased ROS production [43]. Excessive ROS then interacts with polyunsaturated fatty acid phospholipids, leading to lipid peroxidation and changes in cell membranes [44]. Consequently, decreased GSH levels and impaired GPX4 activity can trigger ferroptosis [45]. Tangeretin has been reported to modulate lipid metabolism, as evidenced by its capacity to enhance the hepatic-protective activity of silybin in non-alcoholic steatohepatitis (NASH) [46] and also acted as a regulator of lipoprotein metabolism linked to atherosclerosis development [47]. Furthermore, tangeretin has demonstrated to inhibit fungal ferroptosis in the context of rice blast suppression [27]. Similarly, in our study, we noted that tangeretin significantly restored GPX4 expression and normalized GSH and MDA levels in the lung tissues of mice exposed to LPS. These results suggest that tangeretin inhibits lipid peroxidation and ferroptosis in sepsis-induced lung injury through the inhibition of Xc−/GSH/GPX4 antioxidant axis.

Previous studies have demonstrated that an abnormal inflammatory response is pivotal in iron metabolism disorder and redox system imbalance. Cytokines such as IL-6, IL-1β and TNF-α are known to regulate the synthesis of ferritin, which in turn affects iron storage within cells and tissues [48]. In our study, RNA-seq analysis revealed that the IL6_JAK_STAT3, INFLAMMATORY_RESPONSE and TNF-α_SIGNALING_VIA_NF-κB pathways were suppressed in the LPS + Tan group, and these pathways are closely associated with ferroptosis [49]. Our findings showed that tangeretin significantly decreased ferroptosis in lung tissues and macrophages induced by LPS. Macrophages are pivotal in maintaining immune homeostasis and producing pro-inflammatory factors that facilitate the progression of ALI. Notably, ferroptosis is considered as an immune-derived cell death and is regulated by the immune system. Macrophage ferroptosis has been reported to be involved in chronic obstructive pulmonary disease (COPD) [50], liver ischaemia/reperfusion injury [51], and also sepsis-induced lung injury [34]. The attenuation of macrophage-mediated excessive inflammatory responses has been shown to significantly enhance pulmonary function and improve survival outcomes in sepsis [52, 53]. Consequently, the protective role of tangeretin against sepsis-induced lung injury may be partly due to its anti-inflammatory effect, thus inhibiting macrophage ferroptosis.

Tangeretin has been recognized as an inhibitor of oxidative stress and inflammation, primarily by the upregulation of the Nrf2 signaling pathway in models of collagen-induced arthritis in rats [54] and chromium-induced acute brain injury [16]. Additionally, tangeretin activates Nrf2/antioxidant response element pathway within HEK293T cells [26]. It further sustains antioxidant efficacy by diminishing the ubiquitination of NRF2, which is mediated by CUL3 [15]. In our study, we found that the protective effect of tangeretin on ferroptosis was mediated by activation of Nrf2 signaling pathway. Nrf2, a pivotal regulator of cellular antioxidant responses, is known to confer protection against various oxidative stress-induced forms of cell death, including ferroptosis [55, 56]. This regulatory mechanism involves a gene network related to iron metabolism and lipid peroxidation, underscoring the therapeutic relevance of modulating the Nrf2 pathway in addressing ferroptosis-linked diseases like ALI [25, 57]. Keap1 plays a critical role in this context by forming complexes with Nrf2, leading to its ubiquitination-mediated degradation [58]. Molecular docking results indicated that tangeretin attaches to the apex of the central pore within the Keap1 Kelch domain, with hydrophobic and hydrogen bond interactions facilitating a firm association [26]. Our study highlights that tangeretin treatment significantly upregulates Nrf2 and its downstream target genes HO-1, while downregulates the expression of Keap-1. The upregulation is paralleled by an increase in GSH levels and a simultaneous reduction in MDA levels, observed both in lung tissue and macrophages. Intriguingly, the silencing of Nrf2 expression in macrophages and also in lung tissues abolished the protective role against oxidative stress, inflammatory response, ferroptosis and lung injury induced by LPS. These observations collectively suggest that tangeretin exerts anti-ferroptotic effects by activating the antioxidant defense system, predominantly through the enhancement of Nrf2 expression. Thus, our study not only sheds light on the mechanistic aspects of tangeretin’s action but also highlights its potential as a therapeutic promise for treating diseases associated with ferroptosis.

Additionally, in a mouse model for lung injury caused by sepsis, the application of MCC950 (an NLRP3 inhibitor) inhibited ferroptosis-associated biomarkers, whereas ferrostatin-1 (a ferroptosis inhibitor) led to a decrease in NLRP3 and proteins involved in pyroptosis [59]. This finding suggests a crosstalk between ferroptosis and pyroptosis pathways in lung injury due to sepsis. Research indicates that tangeretin suppress the activation of the NLRP3 inflammasome mediated by ROS [19, 21], indicating the possibility that tangeretin may offer protection against lung injury from sepsis through pyroptosis inhibition and this warrants further exploration. Nevertheless, to reinforce the conclusion of our study, a potential approach involves the genetic knockout of Nrf2 in mice suffering from sepsis-induced lung damage is necessary. Moreover, the mechanism through which tangeretin activates Nrf2 was not explored in this experiment and further study is needed.

## Conclusion

In summary, our study delineates the therapeutic promise of tangeretin in mitigating sepsis-induced ALI, particularly through its inhibition of macrophage ferroptosis facilitated via activation of the Nrf2 signaling pathway. These findings establish a foundation for subsequent investigations into the clinical viability of tangeretin as an innovative treatment strategy for sepsis-induced ALI.

## Supplementary Information

Supplementary Material 1.

## Data Availability

Data will be made available on request.

## Abbreviations

ALI: Acute lung injury
ARDS: Acute respiratory distress syndrome
BALF: Bronchoalveolar lavage fluid
DMEM: Dulbecco’s modified Eagle’s medium
DEGs: Differentially expressed genes
DHE: Dihydroethidium
FBS: Fetal bovine serum
GPX4: Glutathione peroxidase 4
GSH: Glutathione
GSEA: Gene set enrichment analysis
HO-1: Heme oxygenase-1
Keap1: Kelch-like ECH-associated protein 1
LPS: Lipopolysaccharides
MDA: Malondialdehyde
MODS: Multiple organ dysfunction syndrome
MPO: Myeloperoxidase
Nrf2: Nuclear factor erythroid 2-related factor 2
PTGS2: Prostaglandin-endoperoxide synthase 2
ROS: Reactive oxygen species
