# Supplementary material for: Tangeretin alleviates sepsis-induced acute lung injury by inhibiting ferroptosis of macrophage via Nrf2 signaling pathway
Source: Chin Med. 2025 Jan 15;20:11. doi: 10.1186/s13020-025-01063-8 (PMC11734455; doi:10.1186/s13020-025-01063-8)
Supplement: Supplementary file 1 — Supplementary Material 1. [file 13020_2025_1063_MOESM1_ESM.docx]

Supplemental Digital Content

**Tangeretin alleviates sepsis-induced acute lung injury by inhibiting ferroptosis of macrophage via Nrf2 signaling pathway**

Hui Zhang^1,†^, Yan Wang^1,†^, Shenghua Wang^1^, Xiaomei Xue^1^, Kai Huang^1^, Dunfeng Xu^1^, Lai Jiang^1^, Siyuan Li^1,*^, Yunqian Zhang^1,*^

1-Department of Anesthesiology and Surgical Intensive Care Unit, Xinhua Hospital Affiliated to Shanghai Jiao Tong University School of Medicine, Shanghai 200092, P.R.China

^†^These authors contributed equally to this work and should be considered as co-first authors

***Corresponding authors:**

Dr. Siyuan Li

Department of Anesthesiology and Surgical Intensive Care Unit, Xinhua Hospital, Shanghai Jiaotong University School of Medicine, 1665 Kongjiang Road, Shanghai, 200092, China.

Email: [lisiyuan@xinhuamed.com.cn](mailto:lisiyuan@xinhuamed.com.cn)

Dr. Yunqian Zhang

Department of Anesthesiology and Surgical Intensive Care Unit, Xinhua Hospital, Shanghai Jiaotong University School of Medicine, 1665 Kongjiang Road, Shanghai, 200092, China.

Email: [zhangyunqian@xinhuamed.com.cn](mailto:zhangyunqian@xinhuamed.com.cn)

**Material and methods**

**Immunofluorescence staining of lung tissues**

Lung tissues were collected and fixed in 4% paraformaldehyde for further immunofluorescent detection. Lung sections (5 μm) were dewaxed and microwaved in citric acid buffer to retrieve antigens. After blocked with 5% BSA for 30 min, the sections were incubated with anti-F4/80 (1:200, servicebio, #GB11027), anti-CD206 (1:150, R&D Systems, #AF2535), anti-iNOS (1:200, servicebio, # GB125703) and anti-Nrf2 (1:200, servicebio, #GB113808) overnight followed with FITC-conjugated and CY3-conjugated secondary antibody (1:200, servicebio, #GB22303; #GB21303), Nuclei were counterstained with 4’6-diamidino-2-phenylindole (DAPI, Beyotime). Images were taken by Pannoramic MIDI (3D HISTECH, Budapest, Hungary) and analyzed using Image J software.

**RT-PCR**

Total RNA was extracted from lung tissues using TRIzol Reagent. PrimeScript™ RT reagent Kit with gDNA Eraser was used to reverse RNA into cDNA, followed by relative quantitative PCR using ChamQ Universal SYBR qPCR Master Mix. RT-PCR was detected on Real-Time Quantitative PCR instrument (Bio-rad). Primer information was shown in Supplementary Table 1.

**Measurement of lipid ROS in RAW264.7**

Lipid peroxidation was determined using the C11-BODIPY 581/591 fluorescence probe (cat no. S0043, Beyotime) according to the manufacturer's instructions. RAW264.7 cells were treated with different intervention. After harvesting, RAW264.7 were washed and incubated with 2 μM C11-BODIPY 581/591 solution for 20 min. Finally, the images were captured using a fluorescence microscope.

**Cell counting kit-8 (CCK-8) assay**

Cells were seeded in 96-well plates and exposed to the indicated compounds for 12 hours. CCK-8 reagent (cat no. C0038, Beyotime) was added to each well at a 1:10 dilution and incubated for 1 hour to allow the reaction to proceed. Absorbance at 450 nm was then measured using a microplate reader (Bio-Rad, Model 680, USA) to quantitatively evaluate cell viability. All experiments were performed in triplicate to ensure reliability. Ferroptosis inhibitor Ferrostatin-1 (Fer-1, 10 μM, HY-100579), pyroptosis inhibitor VX-765 (10 μM, HY-13205), necroptosis inhibitor Necrostatin-1 (Nec-1, 10 μM，HY-15760), and apoptosis inhibitor Z-VAD-FMK (20 μM, HY-16658B) were obtained from MedChemExpress.

**Supplementary Table 1. Primer Sequences used in this study**

| Gene Name |  | Sequence |
| --- | --- | --- |
| β-actin (F) |  | CTGT ATGC CTCT GGTC GTAC |
| β-actin (R) |  | TGAT GTCA CGCA CGAT TTCC |
| IL-6(F) |  | CTGCAAGAGACTTCCATCCAG |
| IL-6 (R) |  | AGTGGTATAGACAGGTCTGTTGG |
| TNF-α(F) |  | CCTCACCCACACCGTCAG |
| TNF-α(R) |  | GTTGGTCCCCCTTCTCCA |
| IL-1β (F) |  | GAAATGCCACCTTTTGACAGTG |
| IL-1β (R) |  | TGGATGCTCTCATCAGGACAG |
| Arg-1 (F) |  | CTCCAAGCCAAAGTCCTTAGAG |
| Arg-1 (R) |  | AGGAGCTGTCATTAGGGACATC |
| CD206 (F) |  | CTCTGTTCAGCTATTGGACGC |
| CD206 (R) |  | CGGAATTTCTGGGATTCAGCTTC |
| IL-10 (F) |  | GGTTGCCAAGCCTTAGCCAC |
| IL-10 (R) |  | ACCTGCTCCACTGCCTTGCT |

Supplementary Figure 1


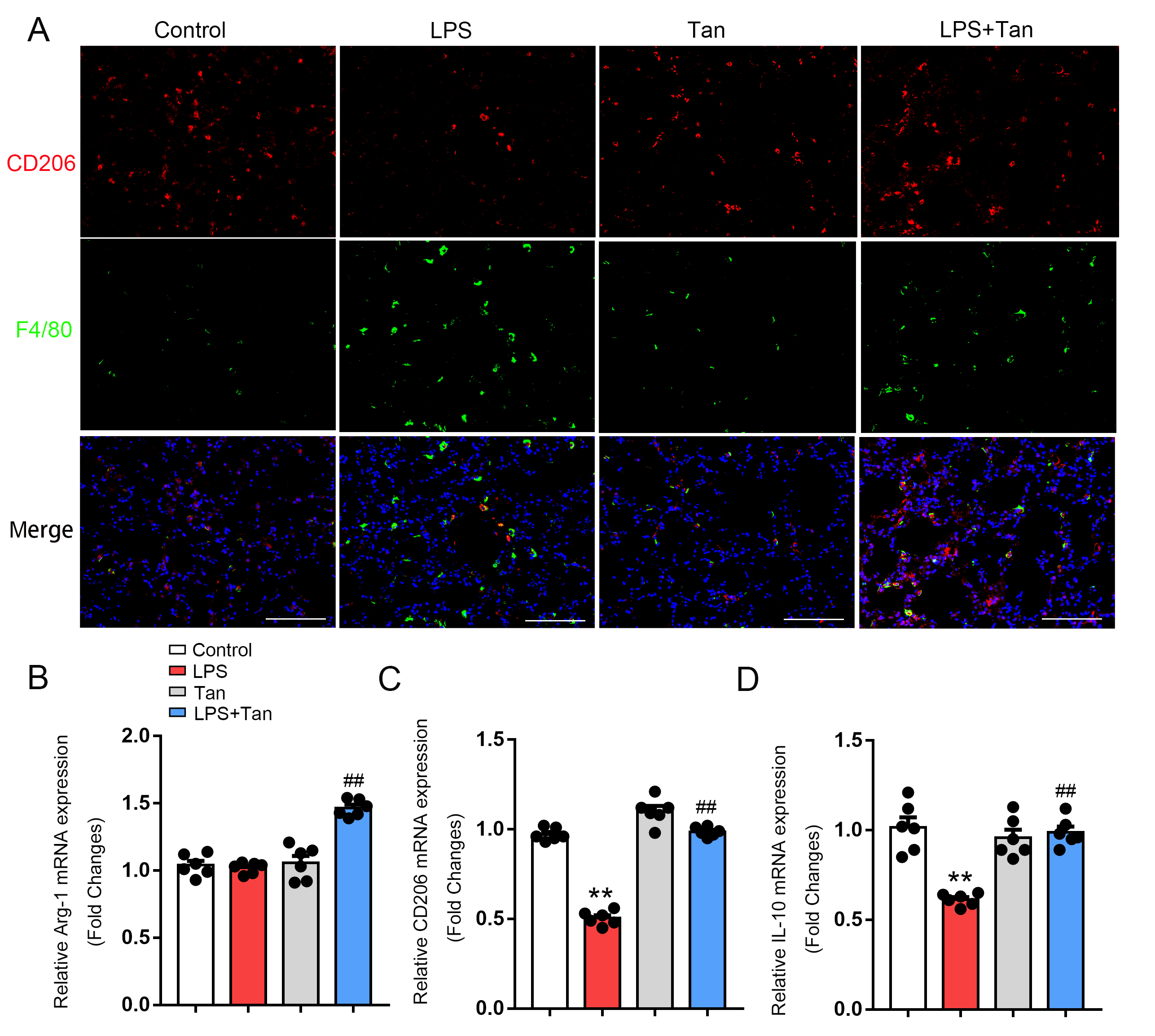


**Supplementary Figure 1. Tangeretin promoted M2 macrophage infiltration in the lung tissues of LPS-induced mice.** Mice were subjected to LPS and treated with tangeretin (i.p). Lung tissues were harvested at the indicated time points. (A) M2 macrophages were shown via double immunofluorescent staining of F4/80 (green) and M2 marker CD206 (red). Nuclei were counterstained with 4′,6-diamidino-2-phenylindole (DAPI) (blue). Merge image represented the staining of F4/80^+^ and CD206^+^ cells in lung tissues. Scale bar = 100 μm. Lung tissues were harvested to examine the relative mRNA expression of M2 markers Arg-1 (B), CD206 (C), and IL-10(D). Data are presented as the mean ± SEM (n = 6). ** p<0.01 vs. Control group; ## p<0.01vs.LPS group.

Supplementary Figure 2


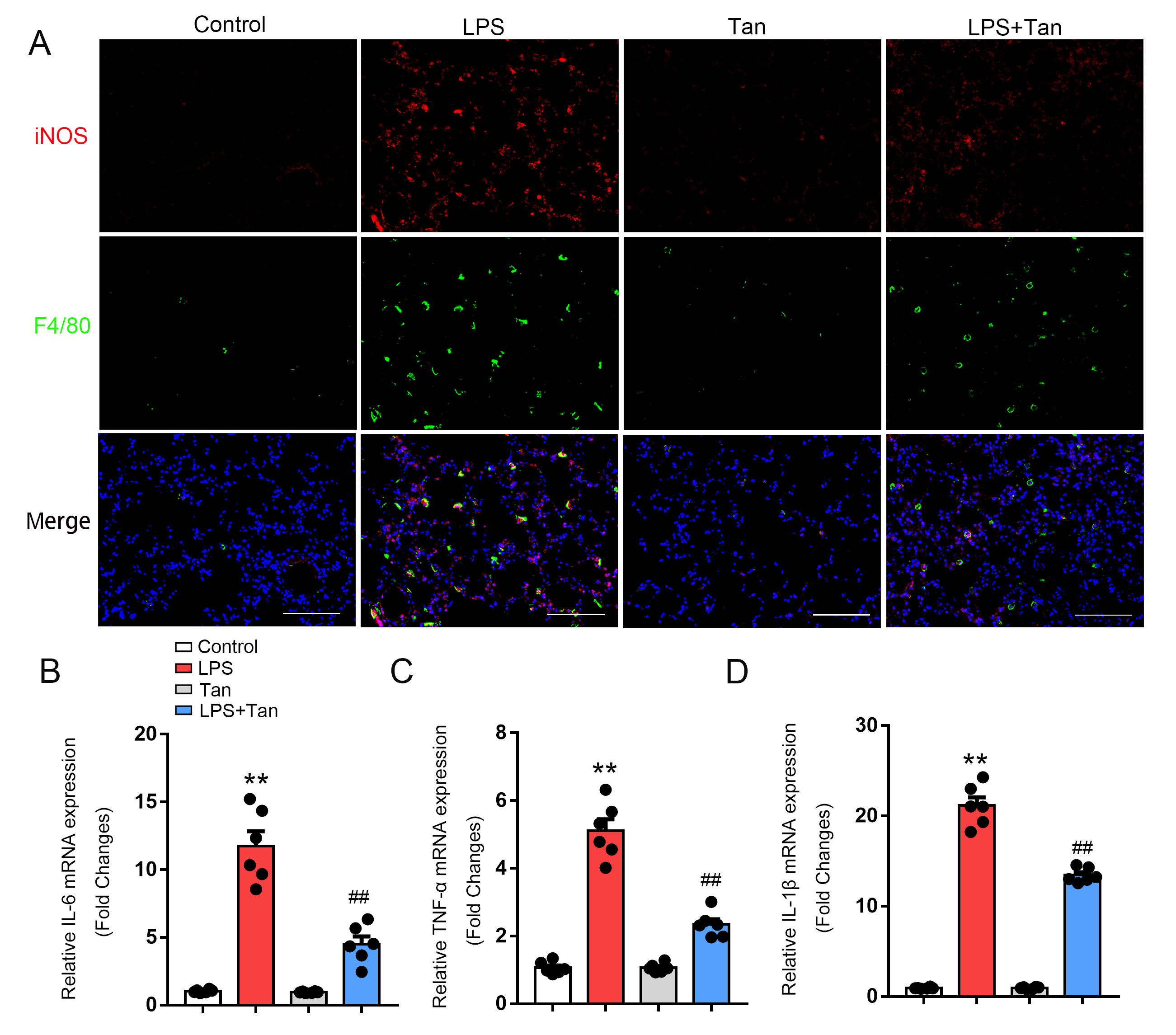


**Supplementary Figure 2. Tangeretin inhibited M1 macrophage infiltration in the lung tissues of LPS-induced mice.** Mice were subjected to LPS and treated with tangeretin (i.p). Lung tissues were harvested at the indicated time points. (A) M1 macrophages were shown via double immunofluorescent staining of F4/80 (green) and M1 marker iNOS (red). Nuclei were counterstained with 4′,6-diamidino-2-phenylindole (DAPI) (blue). Merge image represented the staining of F4/80^+^ and iNOS^+^ cells in lung tissues. Scale bar = 100 μm. Lung tissues were harvested to examine the relative mRNA expression of M1 markers IL-6 (B), TNF-α (C), and IL-1β (D). Data are presented as the mean ± SEM (n = 6). ** p<0.01 vs. Control group; ## p<0.01vs.LPS group.

Supplementary Figure 3


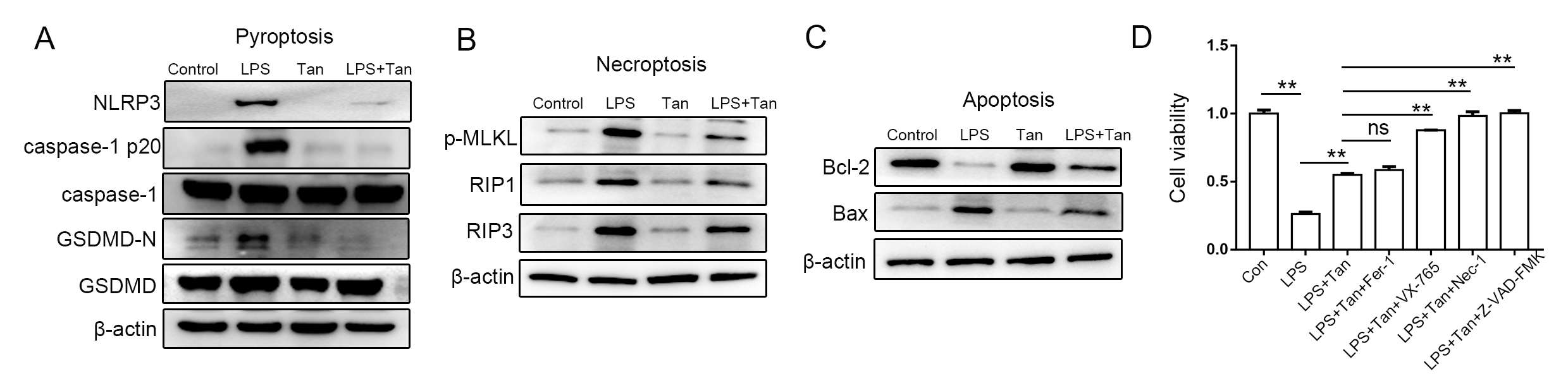


**Supplementary Figure 3. The effect of tangeretin on LPS-induced different forms of cell death.** (A-C) Western blot analyses were conducted to evaluate the expression and activation of key cell death mediators, including NLRP3, cleaved caspase 1 (p20), the N-terminal fragment of GSDMD, p‑MLKL, RIP1, RIP3, Bcl-2, and Bax in RAW 264.7 cells co‑treated with LPS and tangeretin (25 μM). (D) Effects of Fer-1, VX-765, Nec-1, and Z-VAD-FMK on cell viability were assessed by CCK8 assays. Data are presented as the mean ± SEM (n = 4). ** p<0.01 vs. LPS group.

Supplementary Figure 4


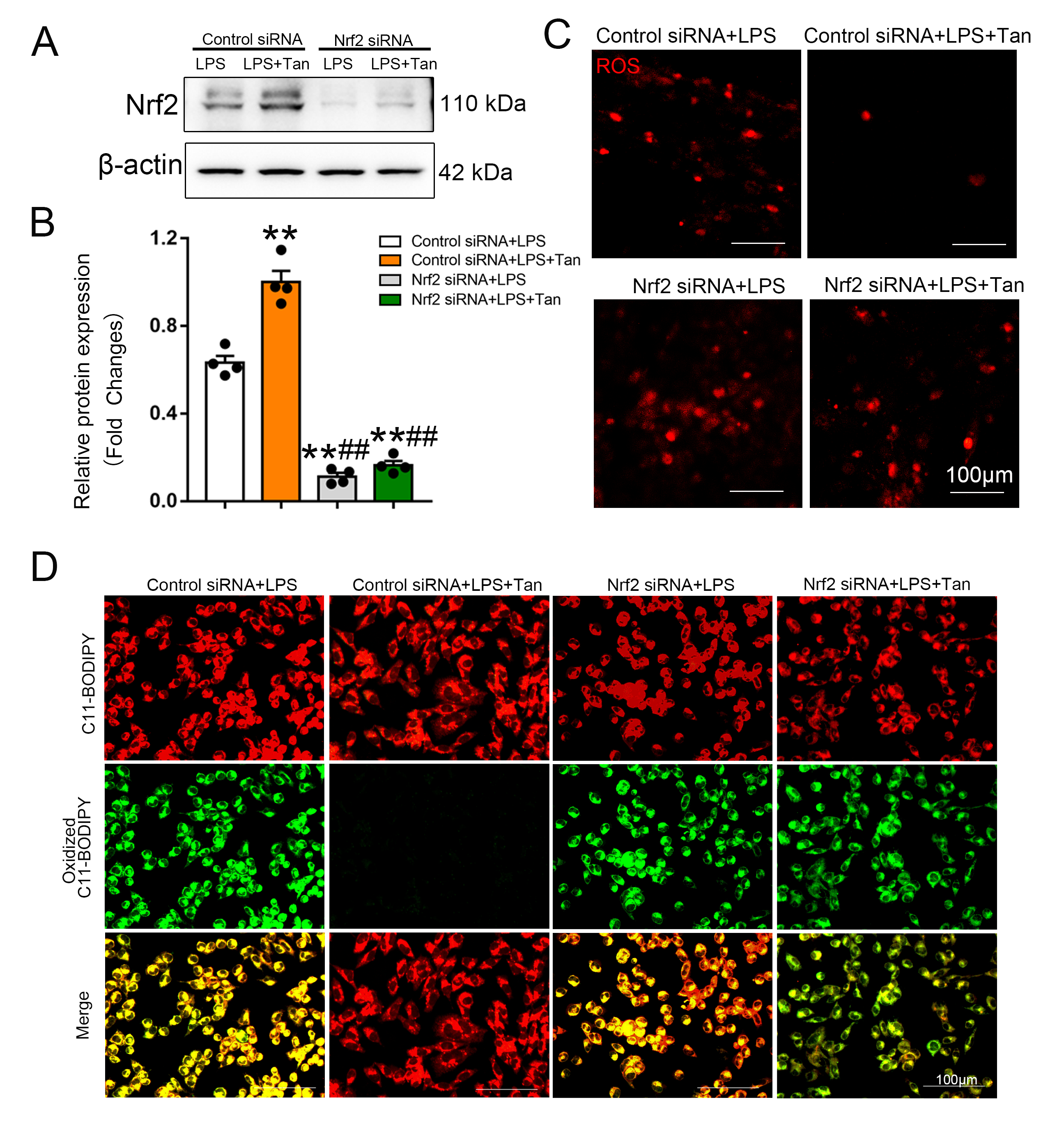


**Supplementary Figure 4. Effect of Nrf2 siRNA on LPS-treated RAW264.7 cell.** RAW264.7 cells transfected with control or Nrf2 siRNA for 48 h, followed by LPS/LPS + Tan treatment. (A) Representative bands and (B) corresponding histograms are shown. Data are presented as the mean ± SEM (n = 4). **p < 0.01 vs. Control siRNA+LPS group, ## p < 0.01 vs. Control siRNA+LPS +Tan group (C) Representative fluorescence probe images of ROS (red). Original magnification, × 200. Scale bar, 100 μm. (D) The representative fluorescent images and quantification of C11-BODIPY probe. The green fluorescence indicated oxidized lipid and the red fluorescence indicated non-oxidized lipid. Original magnification, × 200. Scale bar, 100 μm.

Supplementary Figure 5


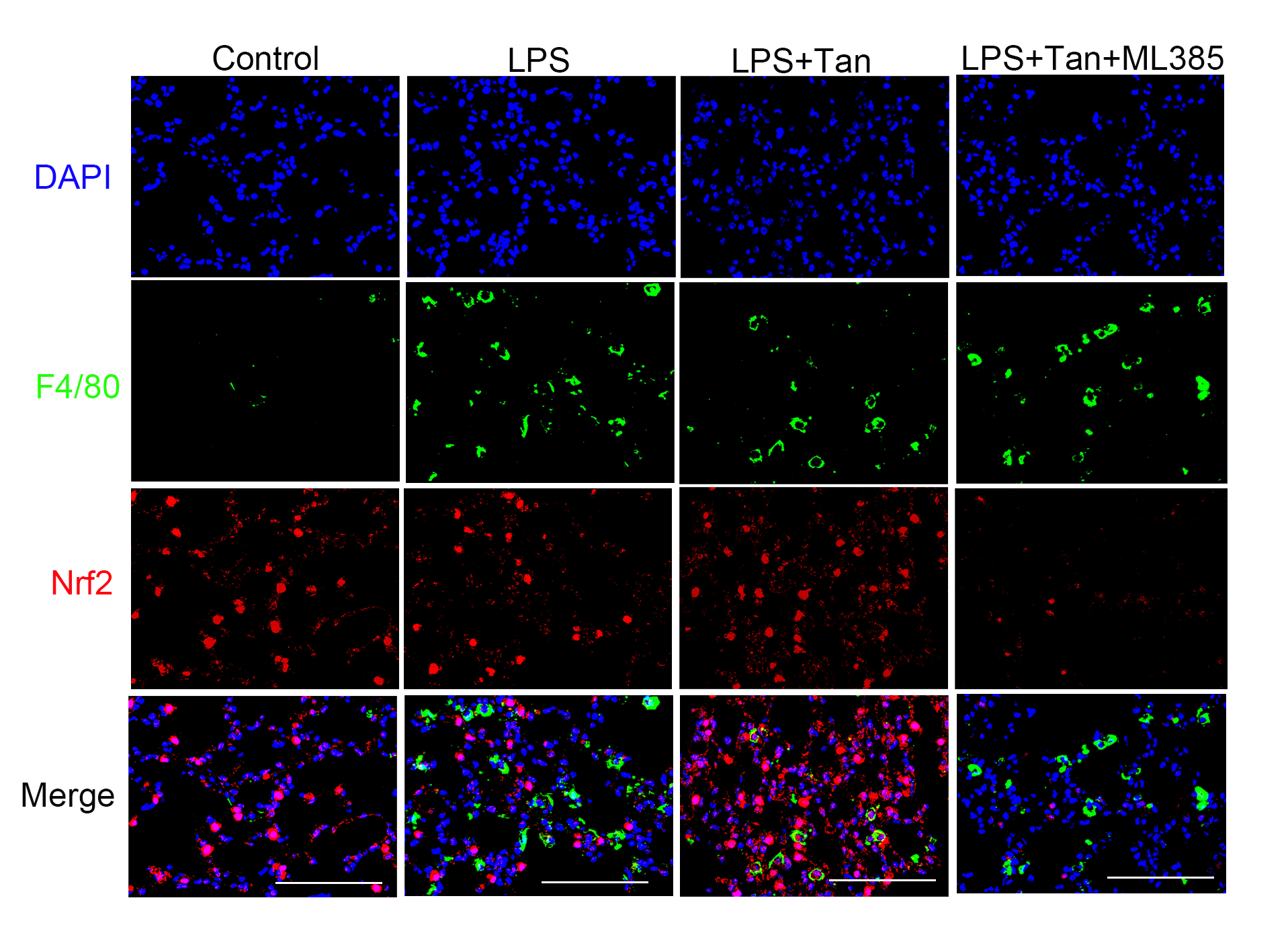


**Supplementary Figure 5. Indentification of the Nrf2 expression in pulmonary macrophage cell.**

ML385 (30 mg/kg) was administered prior to tangeretin treatment. Nrf2 expression in macrophage cell were shown via double immunofluorescent staining of F4/80 (green) and Nrf2 (red). Nuclei were counterstained with 4,6-diamidino-2-phenylindole (DAPI) (blue). Merge image represented the staining of F4/80^+^ and Nrf2^+^ cells in lung tissues. Scale bar = 100 μm.
